# Visualization of the SyncAV^®^ Algorithm for CRT Optimization by Non-invasive Imaging of Cardiac Electrophysiology: NICE-CRT Trial

**DOI:** 10.3390/jcm12134510

**Published:** 2023-07-05

**Authors:** Philipp Spitaler, Bernhard Erich Pfeifer, Agnes Mayr, Rudolf Bachler, Valentin Bilgeri, Agne Adukauskaite, Axel Bauer, Markus Stühlinger, Fabian Barbieri, Wolfgang Dichtl

**Affiliations:** 1 Department of Internal Medicine III, Medical University of Innsbruck, 6020 Innsbruck, Austria; philipp.spitaler@i-med.ac.at (P.S.); valentin.bilgeri@outlook.com (V.B.); agne.adukauskaite@tirol-kliniken.at (A.A.); axel.bauer@i-med.ac.at (A.B.); markus.stuehlinger@tirol-kliniken.at (M.S.); fabian.barbieri@hotmail.com (F.B.); 2 Institute of Clinical Epidemiology, Tirol Kliniken, 6020 Innsbruck, Austria; bernhard.pfeifer@tirol-kliniken.at; 3 Institute of Medical Informatics, UMIT TIROL, Eduart Wallnöfer Zentrum, 6600 Hall in Tirol, Austria; 4 Department of Radiology, Medical University Innsbruck, 6020 Innsbruck, Austria; a.mayr@i-med.ac.at; 5 Abbott Austria, Perfektastrasse 84a, 1230 Wien, Austria; rudolf.bachler@abbott.com; 6 Deutsches Herzzentrum der Charité, Department of Cardiology, Angiology and Intensive Care Medicine, Hindenburgdamm 30, 12203 Berlin, Germany

**Keywords:** fusion pacing, CRT optimization, dynamic AV delay, device programming, left bundle branch block

## Abstract

(1) Background: Periodic repetitive AV interval optimization using a device-based algorithm in cardiac resynchronization therapy (CRT) devices may improve clinical outcomes. There is an unmet need to successfully transform its application into clinical routine. (2) Methods: Non-invasive imaging of cardiac electrophysiology was performed in different device programming settings of the SyncAV^®^ algorithm in 14 heart failure patients with left bundle branch block and a PR interval ≤ 250 milliseconds to determine the shortest ventricular activation time. (3) Results: the best offset time (to be manually programmed) permitting automatic dynamic adjustment of the paced atrioventricular interval after every 256 heart beats was found to be 30 and 50 milliseconds, decreasing mean native QRS duration from 181.6 ± 23.9 milliseconds to 130.7 ± 10.0 and 130.1 ± 10.5 milliseconds, respectively (*p* = 0.01); this was followed by an offset of 40 milliseconds (decreasing QRS duration to 130.1 ± 12.2 milliseconds; *p* = 0.08). (4) Conclusions: The herein presented NICE-CRT study supports the current recommendation to program an offset of 50 milliseconds as default in patients with left bundle branch block and preserved atrioventricular conduction after implantation of a CRT device capable of SyncAV^®^ optimization. Alternatively, offset programming of 30 milliseconds may also be applied as default programming. In patients with no or poor CRT response, additional efforts should be spent to individualize best offset programming with electrocardiographic optimization techniques.

## 1. Introduction

In clinical practice, cardiac resynchronization therapy (CRT) programming is often left at nominal settings after successful implantation, irrespective of individual intrinsic atrioventricular (AV) and/or interventricular (VV) intervals. However, the concept of AV delay (AVD) tailoring by echocardiography and VV delay optimization by electrocardiography (ECG) using the iterative method has long been reported to further improve the positive effects of CRT on reverse remodeling [1].

One innovative alternative approach is to let the device automatically program and repeatedly adjust AV and VV delays based on measurements of the endocardial acceleration with a specific sensor in an atrial lead (SonR tip^®^, Microport^®^, Shanghai, China), but technical issues are still limiting its wider use [2,3].

Other promising device-based algorithms are Adaptive CRT^®^ (Medtronic^®^, Minneapolis, MN, USA) and SyncAV^®^ (Abbott^®^ Laboratories, Abbott Park, IL, USA). While the Adaptive CRT^®^ algorithm does not permit individual programming and ensures dynamic left ventricular (LV) only stimulation without actively pacing through the right ventricular (RV) lead in patients with a PR interval < 200 milliseconds [4], the SyncAV^®^ algorithm favors fusion pacing using a programmable negative AV hysteresis offset with a fixed VV interval (both ventricular leads are stimulating simultaneously after significant parts of the septum and the right ventricle have already been depolarized due to intrinsic conduction).

This SyncAV^®^ algorithm subtracts an offset from the intrinsic AVI which can be individually programmed from 10 to 120 milliseconds by the treating physician. Every 256 beats, the algorithm automatically extends the paced and sensed AV delay (AVD) for three beats, during which it measures the intrinsic AVI and adapts the paced AVD again. Therefore, SyncAV^®^ enables continuous dynamic AVI programming to optimize paced AVD during exercise or changes in autonomic responses, additionally.

The default SyncAV^®^ offset subtracts 50 milliseconds from the intrinsic AVI. An initial study by Varma et al. [5] in 75 patients with left bundle branch block (LBBB) and optimal left ventricular lead position suggests that this default offset shortens QRS to a higher degree than simultaneous biventricular pacing (BIV) with nominal AVD settings (paced/sensed 140/110 milliseconds). However, this study also shows that the optimal offset is not according to a “one size fits all” approach which involves simply programming an offset of 50 milliseconds in every patient but should be individually tailored using surface ECG for optimization. Indeed, the optimal offset was 10, 20, 30, 40, 50, 60 milliseconds in 12, 17.3, 22.6, 14.6, 22.6, 10.6% of patients, respectively.

The NICE-CRT trial was conducted to further validate the best offset programming using non-invasive imaging of cardiac electrophysiology, an emerging imaging tool which works by fusing data from high-resolution electrocardiogram mapping with a model of the patient’s individual cardiothoracic anatomy created from magnetic resonance imaging [6,7,8,9,10,11,12,13].

## 2. Methods

The NICE-CRT study (ClinicalTrials.gov Identifier: NCT04662970) is an investigator-driven prospective study to further assess the best individualized offset to be programmed by the treating physician to maximize the effects of the automatic device-based SyncAV^®^ algorithm. The study protocol was approved by the local ethics committee. The study enrolled patients with New York Heart Association functional class II and III heart failure symptoms, left ventricular ejection fraction < 35%, preserved atrioventricular conduction (resting 12-lead ECG PR interval ≤ 250 milliseconds) and LBBB, while on optimal medical therapy and without permanent atrial tachyarrhythmia. Exclusion criteria were any contraindication to perform cardiac magnetic resonance examination, a PR interval > 250 milliseconds and/or high-grade AV block, terminal heart failure (NYHA IV) or signs of cardiac decompensation, life expectancy < 1 year and women with child-bearing potential, pregnancy or drug abuse.

**Patient characteristics**: Fourteen patients (aged 70 ± 7 years; 78.6% male; ejection fraction 31 ± 6.9%; 50% with ischemic cardiomyopathy) implanted with a CRT device encountering the SyncAV^®^ algorithm (Quadra Assura^®^, CD3367-40QC Quadra Assura MP^®^, CD3371-40QC; Quadra Allure^®^, PM3542; Quadra Allure MP^®^, PM3562; all from Abbott) were evaluated between 26 February 2020 and 24 May 2022. Baseline clinical characteristics are listed in Table 1. All patients were in sinus rhythm with a left bundle branch block at the time of implantation and enrollment. The mean baseline PR interval was 189 ± 36.1 milliseconds and exceeded 200 milliseconds in 6 of 14 (43%) patients. Mean QRS duration was 181.6 ± 23.9 milliseconds. All 14 patients were analyzed in each programming configuration. The SyncAV^®^ algorithm operated effectively in all 14 patients.

**NICE**: Patient specific anatomic parameters taken from the cardiac magnetic resonance examination formed the base for a semiautomatic model incorporating the conductivity of the heart, the lungs, blood and the torso. For this reason, a software package (AMIRA Developer, TGS Template Graphics software version 3.1, Berlin, Germany) has been adapted to calculate a quasi-static approximation of Maxwell equations. After fusion of the T1 CMR scan and the ECG electrodes, a model-based bidomain FEM was used for a step-wise measurement of the local activation times (resting potential: 290 mV; plateau: 0 mV; acceleration time: 3 milliseconds) both from the endo- and the epicardium. Spatial resolution in imaging of cardiac electrical excitation computation typically ranged from 2 to 4 mm, allowing for detailed mapping of the electrical signals within the cardiac tissue [5,6,7,8,9,10,11,12].

**Data acquisition**: NICE was performed during sinus rhythm (mode 1, intrinsic conduction); RV pacing (mode 2, AVD paced 170 milliseconds, sensed 120 milliseconds); nominal = simultaneous biventricular (BIV) pacing (mode 3, AVD paced 170 milliseconds, sensed 120 milliseconds); BIV SyncAV^®^ + offset 10 milliseconds (mode 4); BIV SyncAV^®^ + offset 20 milliseconds (mode 5); BIV SyncAV^®^ + offset 30 milliseconds (mode 6); BIV SyncAV^®^ + offset 40 milliseconds (mode 7); BIV SyncAV^®^ + offset 50 milliseconds (mode 8); BIV SyncAV^®^ + offset 60 milliseconds (mode 9); BIV SyncAV^®^ + offset 70 milliseconds (mode 10); BIV SyncAV^®^ + offset 80 milliseconds (mode 11); BIV SyncAV^®^ + offset 90 milliseconds (mode 12); BIV SyncAV^®^ + offset 100 milliseconds (mode 13); and BIV SyncAV^®^ + offset 110 milliseconds (mode 14). For all pacing modes, right and left ventricular total activation time (VAT); earliest septal, endocardial and epicardial breakthrough sites; and the endocardial/epicardial activation sequences were analyzed.

**Statistics**: Categorical variables are reported as number and percentage, while continuous variables are expressed as mean and standard deviation. Distribution was assessed using the Kolmogorov–Smirnov test and inspection of histograms. Differences in repeated measurements were analyzed with the paired t-test. Statistical analyses were performed using R (version 4.3.1, R-Foundation for Statistical Computing, Vienna, Austria). Graphics designed using ggplot2 package for R. *p* < 0.05 were considered statistically significant.

## 3. Results

**Ventricular activation time: simultaneous biventricular pacing versus intrinsic conduction.** In comparison to intrinsic conduction, simultaneous biventricular pacing reduced the VAT by 40.9 ± 25.7 milliseconds (*p* < 0.001), indicating a relative decrease of 22.5 ± 14.2%.

**Ventricular activation time: optimized SyncAV^®^ versus intrinsic conduction.** In comparison to intrinsic conduction, pacing with optimized SyncAV^®^ reduced the VAT by 63.2 ± 21.2 milliseconds (*p* < 0.001) indicating a relative decrease of 34.8 ± 11.7%.

**Ventricular activation time: optimized SyncAV^®^ versus simultaneous biventricular pacing.** In comparison to simultaneous biventricular pacing, pacing with optimized SyncAV^®^ reduced the VAT by 22.3 ± 17.3 milliseconds (*p* < 0.001) indicating a relative decrease of 15.9 ± 12.3%.

**Figure 1:**
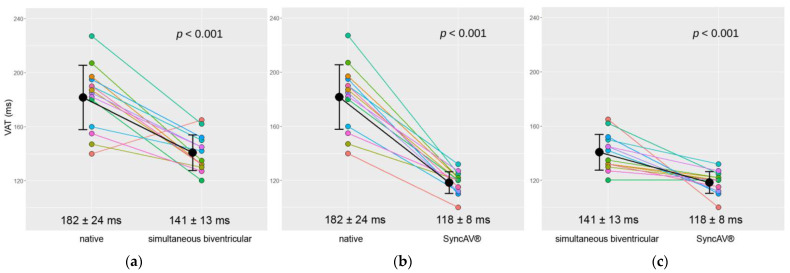
Ventricular activation time (VAT) according to different programming: (**a**) native versus simultaneous biventricular pacing (*p* < 0.001) (**b**) native versus optimized SyncAV^®^ pacing (*p* < 0.001), (**c**) simultaneous biventricular versus optimized SyncAV^®^ pacing (*p* < 0.001).

**Figure 2:**
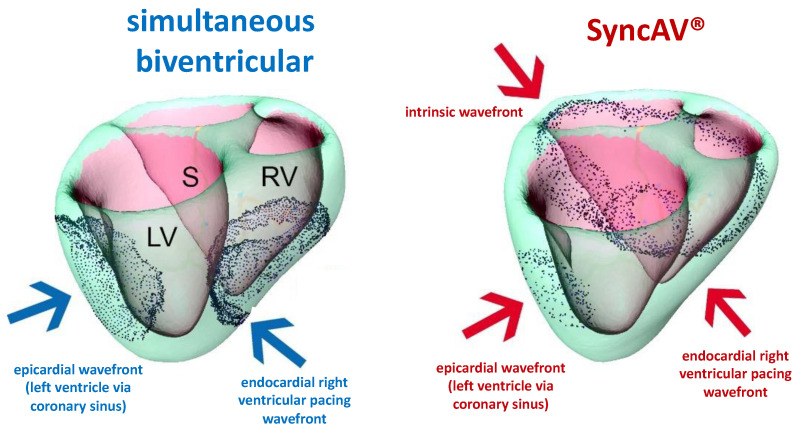
Demonstration of distinct patterns of impulse formation and propagation of simultaneous biventricular pacing versus SyncAV^®^ fusion pacing, respectively. With simultaneous biventricular pacing, two wavefronts originate from each ventricle. In contrast, SyncAV^®^ allows native AV conduction with optimally timed left ventricular fusion pacing, creating a third wavefront and leading to a more physiological and efficient activation of the ventricles (yielding shorter QRS duration as well). Blue arrows: impulse formation and propagation of simultaneous biventricular pacing, red arrows: impulse formation and propagation of SyncAV^®^ fusion pacing. LV = left ventricle, RV = right ventricle, S = septum.

**Figure 3:**
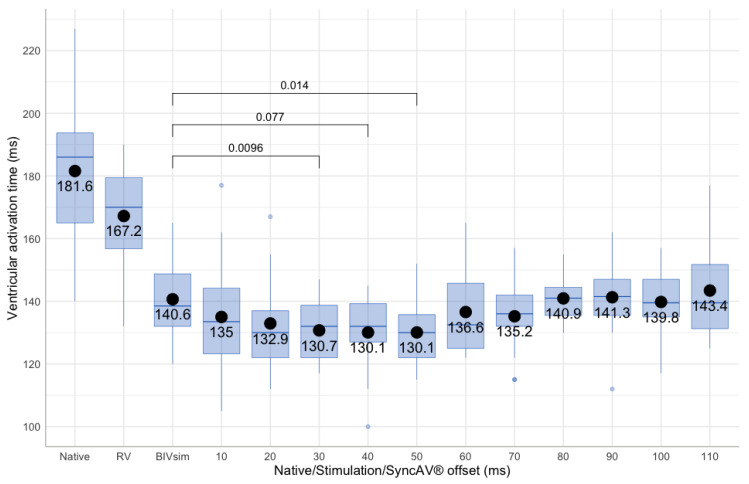
Mean ventricular activation time (VAT) and its dependency on different programming in the study cohort.

**Figure 4:**
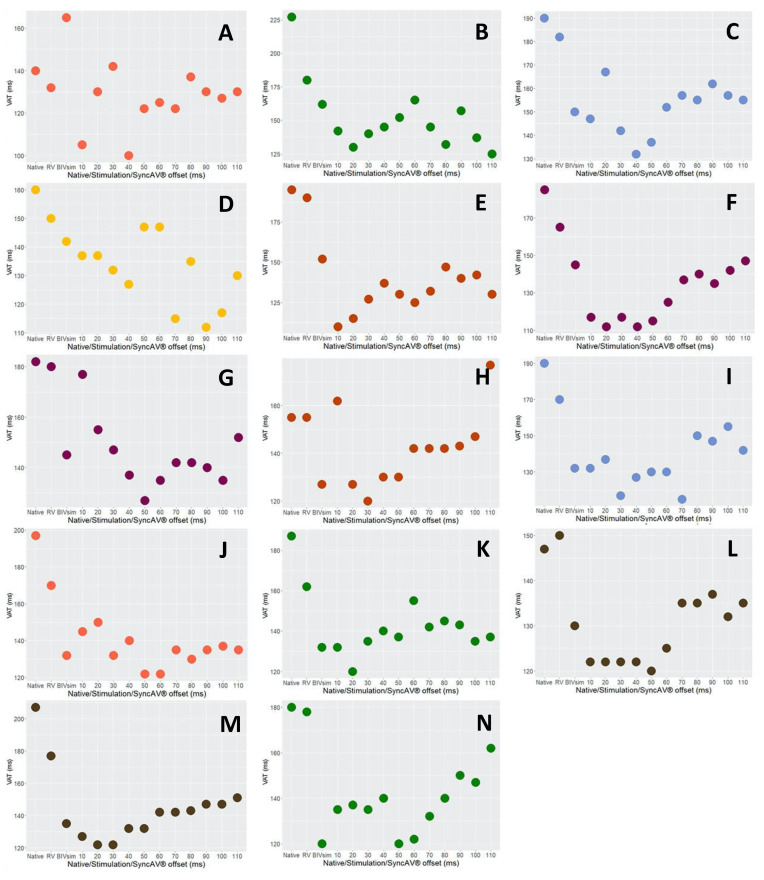
Ventricular activation time (VAT) and its dependency from different programming—data from the 14 individual (**A**–**N**) cases of the study cohort.

**Figure 5:**
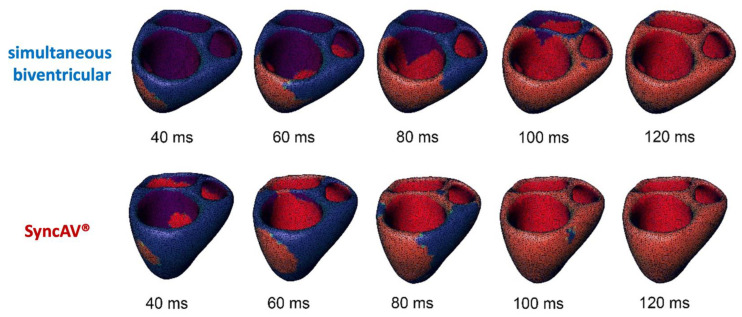
Ventricular activation visualized by red color, lateral view (left ventricle is in front; right ventricle is in the back), ms denotes milliseconds.

**Figure 6:**
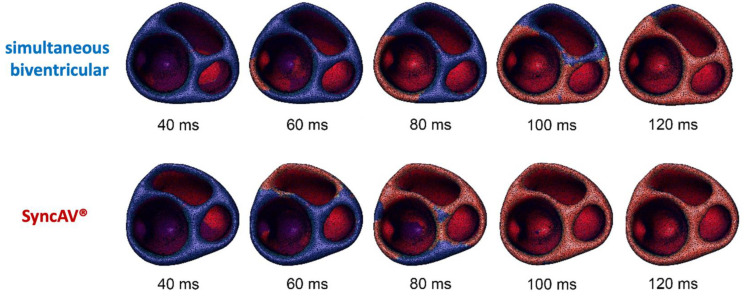
Ventricular activation visualized by red color, transversal view (left ventricle is left/in the front; right ventricle is right/in the back); ms denotes milliseconds.

Individual results from the whole study cohort are shown in Figure 1, Figure 3 and Figure 4. Summary comparisons can be found in Table 2. A representative example case is illustrated in Figure 2, Figure 5 and Figure 6, comparing simultaneous biventricular pacing to SyncAV^®^ fusion pacing in an individual patient (labeled B in Figure 4; see also Video S1 in Supplementary File).

## 4. Discussion

Structural or clinical responses to CRT are not binary but continuous phenomena, and optimal postimplant device programming may finally improve originally labeled “nonresponders” and turn “responders” to “super-responders”. From the infancy of this therapeutic approach more than two decades ago, the importance of the optimal AVD programming has been acknowledged but the original approach optimizing transmitral flow characteristics and/or stroke volume by echocardiography is time-consuming, operator-dependent and overall cumbersome. Therefore, echocardiography-guided CRT optimization has hardly become clinical routine from a global perspective. Because of these shortcomings, device-based automatic AVD programming has been introduced, with the SyncAV^®^ algorithm being one of the most recently developed concepts. SyncAV^®^ fusion pacing provides an improvement to acute hemodynamic measures and reverse remodeling on echocardiography [14,15].

In our view, SyncAV^®^ represents a golden mean between simultaneous biventricular pacing and left ventricular only pacing, offering a practical opportunity easily applied in clinical routine. Simultaneous biventricular pacing strongly reduces interventricular dyssynchrony by minimizing fusion and altering left and right ventricular activation times to a common level, as recently demonstrated using ECGi yielding reduced ventricular uncoupling index measurements [16]. However, simultaneous biventricular pacing often results in only modest QRS duration reductions, reflecting a lost opportunity for further resynchronization with residual intraventricular dyssynchrony.

LV only pacing, going in the opposite direction, has the tendency to change the lateral wall from being the latest to the earliest point of ventricular activation, therefore turning around the wavefront of dyssynchrony. LV only pacing typically results in a right bundle branch block (RBBB) QRS pattern with a dominant R wave in lead V_1_ (and often a negative QRS in lead I). In the B-LEFT study, LV only pacing has been shown equivalence as compared with biventricular pacing, maybe even favoring LV reverse remodeling [17,18]. LV only pacing may therefore be an alternative in previous nonresponders to biventricular pacing as shown in the GREATER EARTH study [19].

NICE-CRT exclusively tested patients with LBBB, while results with non-LBBB configurations were not assessed. In particular, RBBB patients usually need longer optimal offsets, around 90 milliseconds [20]. Given that the AVD is measured by the device using the right ventricular lead, the presence of RBBB results in the delayed detection of ventricular activation (relative to surface ECG) and the need to program a more negative AV offset to achieve fusion. In some but not all patients, MultiPoint pacing (MPP) added to the SyncAV^®^ algorithm further increased resynchronization, as recently shown by ECGi [16,21,22].

The large, randomized trial ADAPT RESPONSE has recently shown a (nonsignificant) 11% reduction of a combined endpoint of intervention for heart failure decompensation or all-cause mortality during long-term follow-up in patients with heart failure, reduced left ventricular ejection fraction (<35%), LBBB and intact AV conduction (a patient cohort exactly matching patients enrolled in the herein presented NICE-CRT trial) [23]. The subgroup of patients with ≥85% synchronized LV pacing has shown a significantly (24%) lower mortality/heart failure rate. The SyncAV^®^ algorithm is expected to improve clinical outcomes in CRT patients to the same or even higher extent with a high likelihood.

The results of the herein presented NICE-CRT study show that the SyncAV^®^ offset should be programmed to 30–50 milliseconds in the majority of patients with a native LBBB and a PR interval ≤ 250 milliseconds. For practical reasons, the current recommendation of 50 milliseconds as default programming seems to be an appropriate starting point. However, at least in patients with no or poor CRT response, additional efforts should be spent to individualize device programming using electrocardiographic optimization techniques, aiming to reach the best shortening of QRS duration (or QRS area reduction). These approaches include recording and comparing 12-lead electrocardiograms in different programming settings (for example, with offsets at 20, 30, 40, 50 and 60 milliseconds). A strategy which can also be applied in devices from other vendors, without using the SyncAV^®^ algorithm, is referred to as fusion-optimized intervals [24,25]. In research or selected clinical cases, more advanced but also more expensive and time-consuming methods such as body surface activation mapping (for example, with ECGi) or, as presented here for the first time with the NICE-CRT trial, non-invasive imaging of cardiac electrophysiology play an emerging role ([26], for review).

## 5. Limitations

The number of patients enrolled in the NICE-CRT trial is small and the data should be interpretated as a single-center pilot study. Mean native QRS duration in NICE-CRT (181.6 ± 23.9 milliseconds) was longer than in many CRT trials. For example, QRS duration was 162.6 ± 17.1 milliseconds in the intervention arm of the ADAPT-CRT trial analyzing 1810 patients [23]. The beneficial effects of the SyncAV^®^ algorithm might be lower in patients with shorter native QRS prolongation. In general, a reduction in QRS duration is only a proxy for CRT response, and other measures such as QRS area reduction might become reasonable alternatives for optimizing CRT programming in future clinical routine [27].

## 6. Conclusions

The NICE-CRT trial visualizes and further validates the concept that dynamic AV delay programming in CRT patients targeting fusion with intrinsic conduction further increases resynchronization compared to simultaneous biventricular pacing. It seems worth making the effort to program SyncAV^®^ at an individualized offset. It highlights the profound need for health care professionals responsible for device programming and follow-up monitoring to become acquainted with this algorithm.

## Figures and Tables

**Table 1:** Baseline clinical characteristics.

	*n* = 14
Age, years, mean	70 ± 7
Male gender, *n* (%)	11 (78.6)
Ischemic cardiomyopathy, *n* (%)	7 (50.0)
Left ventricular ejection fraction, %, mean	31 ± 6.9
PR interval, milliseconds, mean	189.0 ± 36.1
Native QRS duration, milliseconds, mean	181.6 ± 23.9
Arterial hypertension, *n* (%)	9 (64.3)
Diabetes mellitus Type II, *n* (%)	5 (35.7)

**Table 2:** Ventricular activation time (VAT), its dependency from different programming and comparison of SyncAV^®^ to biventricular stimulation; ms denotes milliseconds.

	VAT (ms)	Absolute Difference to Biventricular Stimulation (ms)	95% Confidence Interval	*p*
Native conduction, ms, mean	181.6 ± 23.9	+40.9	26.1 to 55.8	<0.001
Right ventricular stimulation, ms, mean	167.2 ± 16.0	+26.6	14.4 to 38.8	<0.001
Simultaneous biventricular, ms, mean	140.6 ± 13.3	0	0	1
SyncAV^®^, offset 10 ms, ms, mean	135.0 ± 19.4	−5.6	−20.8 to −9.5	0.434
SyncAV^®^, offset 20 ms, ms, mean	132.9 ± 15.7	−7.7	−19.9 to 4.0	0.177
SyncAV^®^, offset 30 ms, ms, mean	130.7 ± 10.0	−9.9	−17.1 to −2.9	0.010
SyncAV^®^, offset 40 ms, ms, mean	130.1 ± 12.2	−10.6	−22.5 to 1.3	0.077
SyncAV^®^, offset 50 ms, ms, mean	130.1 ± 10.5	−10.6	−18.6 to −2.5	0.014
SyncAV^®^, offset 60 ms, ms, mean	136.6 ± 14.0	−4.1	−13.6 to 5.0	0.375
SyncAV^®^, offset 70 ms, ms, mean	135.2 ± 11. 7	−5.4	−15.3 to 4.5	0.257
SyncAV^®^, offset 80 ms, ms, mean	140.9 ± 6.9	+0.3	−8.5 to 9.1	0.945
SyncAV^®^, offset 90 ms, ms, mean	141.3 ± 12.1	+0.6	−9.8 to 11.1	0.896
SyncAV^®^, offset 100 ms, ms, mean	139.8 ± 10.7	−0.9	−11.9 to 10.2	0.870
SyncAV^®^, offset 110 ms, ms, mean	143.4 ± 14.7	+2.8	−11.4 to 17.0	0.679

## Data Availability

All data are available on a reasonable request to the corresponding author.

## Supplementary Materials

The following are available online at https://www.mdpi.com/article/10.3390/jcm12134510/s1, Video S1: NICE CRT clips.

## References

[1] Bertini M., Ziacchi M., Biffi M., Martignani C., Saporito D., Valzania C., Diemberger I., Cervi E., Frisoni J., Sangiorgi D. (2008). Interventricular delay interval optimization in cardiac resynchronization therapy guided by echocardiography versus guided by electrocardiographic QRS interval width. Am. J. Cardiol..

[2] Brugada J., Delnoy P.P., Brachmann J., Reynolds D., Padeletti L., Noelker G., Kantipudi C., Robin Lopez J.M., Dichtl W., Borri-Brunetto A. (2017). Contractility Sensor Guided Optimization of Cardiac Resynchronization Therapy–Results from the RESPOND-CRT trial. Eur. Heart J..

[3] Senoner T., Barbieri F., Semmler G., Adukauskaite A., Rubatscher A., Schgör W., Stühlinger M., Bauer A., Pfeifer B.E., Fiedler L. (2019). Long-term performance of an atrial lead capable of accelerometer based detection of cardiac contractility in patients receiving cardiac resynchronisation therapy. PLoS ONE.

[4] Martin D.O., Lemke B., Birnie D., Krum H., Lee K.L., Aonuma K., Gasparini M., Starling R.C., Milasinovic G., Rogers T. (2012). Investigation of a novel algorithm for synchronized left-ventricular pacing and ambulatory optimization of cardiac resynchronization therapy: Results of the adaptive CRT trial. Heart Rhythm.

[5] Varma N., O’Donnell D., Bassiouny M., Ritter P., Pappone C., Mangual J., Cantillon D., Badie N., Thibault B., Wisnoskey B. (2018). Programming cardiac resynchronization therapy for electrical synchrony: Reaching beyond left bundle branch block and left ventricular activation delay. J. Am. Heart Assoc..

[6] Modre R., Tilg B., Fischer G., Hanser F., Messnarz B., Seger M., Schocke M.F., Berger T., Hintringer F., Roithinger F.X. (2003). Atrial noninvasive activation mapping of paced rhythm data. J. Cardiovasc. Electrophysiol..

[7] Tilg B., Fischer G., Modre R., Hanser F., Messnarz B., Schocke M., Kremser C., Berger T., Hintringer F., Roithinger F.X. (2002). Model-based imaging of cardiac electrical excitation in humans. IEEE Trans. Med. Imaging.

[8] Tilg B., Fischer G., Modre R., Hanser F., Messnarz B., Roithinger F.X. (2003). Electrocardiograohic imaging of atrial and ventricular activation. Med. Image Anal..

[9] Berger T., Fischer G., Pfeifer B., Modre R., Hanser F., Trieb T., Roithinger F.X., Stuehlinger M., Pachinger O., Tilg B. (2006). Single-beat noninvasive imaging of cardiac electrophysiology of ventricular pre-excitation. J. Am. Coll. Cardiol..

[10] Berger T., Hanser F., Hintringer F., Poelzl G., Fischer G., Modre R., Tilg B., Pachinger O., Roithinger F.X. (2005). Effects of cardiac resynchronization therapy on ventricular repolarization in patients with congestive heart failure. J. Cardiovasc. Electrophysiol..

[11] Berger T., Pfeifer B.E., Hanser F.F., Hintringer F., Fischer G., Netzer M., Trieb T., Stuehlinger M., Dichtl W., Baumgartner C. (2011). Single-Beat Noninvasive Imaging of Ventricular Endocardial and epicardial Activation in Patients Undergoing CRT. PLoS ONE.

[12] Seger M., Hanser F., Dichtl W., Stühlinger M., Hintringer F., Trieb T., Pfeifer B., Berger T. (2014). Non-Invasive Imaging of Electrical Excitation in a CRT-D Patient with a Quadripolar Left Ventricular Lead. Europace.

[13] Barbieri F., Pfeifer B., Berger T., Dichtl W. (2015). Comparison of Conventional Resynchronization to Multipoint Pacing Using Two Seperate Left Ventricular Leads by Non-Invasive Imaging of Cardiac Electrophysiology. Eur. Heart J..

[14] Wang J., Liang Y., Chen H., Wang W., Bai J., Chen X., Qin S., Su Y., Ge J. (2020). Patient-tailored SyncAV algorithm: A novel strategy to improve synchrony and acute hemodynamic response in heart failure patients treated by cardiac resynchronization therapy. J. Cardiovasc. Electrophysiol..

[15] Thibault B., Ritter P., Bode K., Calò L., Mondésert B., Mangual J.O., Badie N., McSpadden L.C., Pappone C., Varma N. (2019). Dynamic programming of atrioventricular delay improves electrical synchrony in a multicenter cardiac resynchronization therapy study. Heart Rhythm.

[16] Waddingham P.H., Mangual J.O., Orini M., Badie N., Muthumala A., Sporton S., McSpadden L.C., Lambiase P.D., Chow A.W.C. (2023). Electrocardiographic imaging demonstrates electrical synchrony improvement by dynamic atrioventricular delays in patients with left bundle branch block and preserved atrioventricular conduction. Europace.

[17] Boriani G., Kranig W., Donal E., Calo L., Casella M., Delarche N., Lozano I.F., Ansalone G., Biffi M., Boulogne E. (2010). A randomized double-blind comparison of biventricular versus left ventricular stimulation for cardiac resynchronization therapy: The Biventricular versus Left Univentricular Pacing with ICD Back-up in Heart Failure Patients (B-LEFT HF) trial. Am. Heart J..

[18] Ansalone G., Boriani G., Sassone B., Camastra G., Donal E., Calò L., Casella M., Delarche N., Lozano I.F., Biffi M. (2023). Biventricular versus left ventricular only stimulation: An echocardiographic substudy of the B-LEFT HF trial. J. Cardiovasc. Med..

[19] Thibault B., Ducharme A., Harel F., White M., O’Meara E., Guertin M.C., Lavoie J., Frasure-Smith N., Dubuc M., Guerra P. (2011). Left ventricular versus simultaneous biventricular pacing in patients with heart failure and a QRS complex ≥120 milliseconds. Circulation.

[20] AlTurki A., Lima P.Y., Vidal A., Toscani B., Diaz S., Garcia D., Montemezzo M., Al-Dossari A., Bernier M.L., Hadjis T. (2021). Fusion pacing in patients with right bundle branch block who undergo cardiac resynchronization therapy. J. Electrocardiol..

[21] Schiedat F., Mijic D., Karosiene Z., Bogossian H., Zarse M., Lemke B., Hanefeld C., Mügge A., Kloppe A. (2021). Improvement of electrical synchrony in cardiac resynchronization therapy using dynamic atrioventricular delay programming and multipoint pacing. Pacing Clin. Electrophysiol..

[22] O’Donnell D., Wisnoskey B., Badie N., Odgers L., Smart T., Ord M., Lin T., Mangual J.O., Cranke G., McSpadden L.C. (2021). Electrical synchronization achieved by multipoint pacing combined with dynamic atrioventricular delay. J. Interv. Card Electrophysiol..

[23] Wilkoff B.L., Birnie D., Leclercq C., Gold M.R., Hersi A.S., Kusano K., Mullens W., Gerritse B., van Wel J., Filippatos G. Adaptive versus conventional cardiac resynchronization therapy in patients with heart failure. Lancet.

[24] Arbelo E., Tolosana J.M., Trucco E., Penela D., Borràs R., Doltra A., Andreu D., Aceña M., Berruezo A., Sitges M. (2014). Fusion-optimized intervals (FOI): A new method to achieve the narrowest QRS for optimization of the AV and VV intervals in patients undergoing cardiac resynchronization therapy. J. Cardiovasc. Electrophysiol..

[25] Pujol-López M., Tolosana J.M., Guasch E., Trucco E., Jiménez-Arjona R., Borràs R., Garre P., San Antonio R., Doltra A., Roca-Luque I. (2021). Cardiac Resynchronization Therapy Response Is Equalized in Men and Women by Electrical Optimization: PR Matters. JACC Clin. Electrophysiol..

[26] Pujol-López M., San Antonio R., Mont L., Trucco E., Tolosana J.M., Arbelo E., Guasch E., Heist E.K., Singh J.P. (2019). Electrocardiographic optimization techniques in resynchronization therapy. Europace.

[27] van Stipdonk A.M.W., Ter Horst I., Kloosterman M., Engels E.B., Rienstra M., Crijns H.J.G.M., Vos M.A., van Gelder I.C., Prinzen F.W., Meine M. (2018). QRS Area Is a Strong Determinant of Outcome in Cardiac Resynchronization Therapy. Circ. Arrhythm. Electrophysiol..

